# Retraction of “Novel Bioluminescent Activatable Reporter for Src Tyrosine Kinase Activity in Living Mice”

**DOI:** 10.7150/thno.52610

**Published:** 2020-09-30

**Authors:** Weibing Leng, Dezhi Li, Liang Chen, Hongwei Xia, Qiulin Tang, Baoqin Chen, Qiyong Gong, Fabao Gao, Feng Bi

**Affiliations:** 1 Department of Medical Oncology, West China Hospital, Sichuan University, Chengdu 610041, Sichuan, China; 2 Laboratory of Signal Transduction & Molecular Targeted Therapy, State Key Laboratory of Biotherapy, Sichuan University, Chengdu 610041, Sichuan, China; 3 Department of Radiology, West China Hospital, Sichuan University, Chengdu 610041, Sichuan, China

The Editor-In-Chief of Theranostics, in consultation and agreement with the editorial board members, retracts the article “Novel Bioluminescent Activatable Reporter for Src Tyrosine Kinase Activity in Living Mice” 1 on the basis of questions related to the Western blots within several figures. The concerns about the figures also raise questions about the conclusions within the paper.
